# 921 How to Become a Burn Nurse

**DOI:** 10.1093/jbcr/iraf019.452

**Published:** 2025-04-01

**Authors:** Anita Au, Ashley Callahan, Stephanie Mason

**Affiliations:** Sunnybrook Health Sciences Centre; Ross Tilley Burn Centre, Sunnybrook Health Sciences Centre; Ross Tilley Burn Centre, Sunnybrook Health Sciences Centre

## Abstract

**Introduction:**

For ninth consecutive year, CIHI reported the province has the worst RN to population ratio in the country, needing 26,000 additional nurses. With shortage of ICU nurses, staff continue experiencing burnout, the ongoing impact of the pandemic recovery experience, and the perception of a lack of team cohesiveness. As a provincial unit with 14 beds, staffing dropped from 46 to 15 full/part-time staff during COVID. Half of the nurses have less than 2 years of experience. Immediate action needs to be taken. The goal is to create an education pathway based on the American Burn Competencies Standards and the Standards for Critical Care Nursing Practice that adapts to individualized learning style as part of recruitment and retention strategy.

**Methods:**

A roadmap using the Prosci Methodology is used by the newly recruited nurses to follow as they adopt new behaviors or ways of thinking. The effectiveness of the educational pathway is assessed using the PDSA cycle. Educational burn topics are established based on the certified burn registered nurse certificate. Nurses are from different levels of nursing experience or non-experience (new graduate, with or without ICU, with or without Burn). Using adult self-learning theory, the five senses guide the learning milestone: e-modules, “show and tell” clinical practice, games, return demonstrations, simulation labs of scenarios, test and quizzes, micro-teaching sessions, buddy shifts, self-reflection goals and achieving specializing certificates like ABLS, ACLS, CCNC, CBRN.

**Results:**

There is a continuous demand for ICU services while units have ICU nurses’ shortages. Nurses attend the critical care sponsorship program to obtain basic ICU skills, but no specific program provides the burn specialty skills. By developing an educational pathway, new burn staff are supported from beginning to two years after being hired. For retention, a standard of burn competency is established that encourages professional development. A cohesive regional burn team is the result.

**Conclusions:**

There is a continuous demand for ICU services while units are experiencing ICU nursing shortages. Nurses attend the critical care sponsorship program to obtain basic ICU skills, but no specific program provide the burn specialty skills. By developing an educational pathway, new burn staff are supported from beginning to two years after being hired. For retention, a standard of burn competency is established that encourages professional development. A cohesive regional burn team is the result.

**Applicability of Research to Practice:**

As part of the discussion, this project is able to inspire nurses to join the team and to retain nurses in the burn ICU specialization while enable the creation of a supportive environment that nurses feel ready for professional development in an intensive burn care through completing an environmental safety survey and participation in national or international ICU or burn conferences.

**Funding for the Study:**

N/A

